# *N-*phenyl pyrazoline derivative inhibits cell aggressiveness and enhances paclitaxel sensitivity of triple negative breast cancer cells

**DOI:** 10.1038/s41598-024-63778-2

**Published:** 2024-06-08

**Authors:** Pamungkas Bagus Satriyo, Mustofa Mustofa, Tutik Dwi Wahyuningsih, Ema Damayanti, Hesti Lina Wiraswati, Denny Satria, M. Hasan Bashari, Eti Nurwening Sholikhah

**Affiliations:** 1 Department of Pharmacology and Therapy, Faculty of Medicine Public Health and Nursing, Universitas Gadjah Mada, Yogyakarta, 55281 Indonesia; 2 Department of Chemistry, Faculty of Mathematics and Natural Sciences, Universitas Gadjah Mada, Yogyakarta, 55281 Indonesia; 3 Research Center for Food Technology and Processing, National Research and Innovation Agency, Gunungkidul, 55861 Indonesia; 4 Department of Biomedical Sciences, Faculty of Medicine, Universitas Padjadjaran, Sumedang, Indonesia; 5 Department of Pharmaceutical Biology, Faculty of Pharmacy, Universitas Sumatera Utara, Medan, Indonesia; 6 Oncology and Stem Cell Working Group, Faculty of Medicine, Universitas Padjadjaran, Bandung, Indonesia

**Keywords:** *N*-phenyl pyrazoline, Triple-negative breast cancer, In vitro, In silico, Cancer, Drug discovery

## Abstract

Protein kinase dysregulation induces cancer cell aggressiveness leading to rapid tumor progression and poor prognosis in TNBC patients. Many small-molecule kinase inhibitors have been tested in clinical trials to treat TNBC patients. In the previous study, we found that *N*-phenylpyrazoline small molecule acts as a protein kinase inhibitor in cervical cancer cells. However, there remains unknown about *N*-phenyl pyrazoline potency as a kinase inhibitor and its anti-cancer activity in TNBC cells. In this study, we investigated the activity of *N*-phenyl pyrazoline against TNBC cells via tyrosine kinase inhibition. Based on the MTT assay, the IC50 values for the *N*-phenyl pyrazoline 2, 5, A, B, C, and D against Hs578T were 12.63 µM, 3.95 µM, not available, 18.62 µM, 30.13 µM, and 26.79 µM, respectively. While only P5 exhibited the IC50 against MDA MB 231 (21.55 µM). Further, *N*-phenyl pyrazoline 5 treatment significantly inhibited the cell proliferation rate of Hs578T and MDA MB 231 cells. The migration assay showed that treatment with the compound *N*-phenyl pyrazoline 5 with 4 µM concentration significantly reduced cell migration of Hs578T cells. *N*-phenyl pyrazoline 5 treatment at 1 µM and 2 µM was able to reduce the tumorsphere size of Hs578t cells. A combination treatment of P5 and paclitaxel showed a synergistic effect with a combination index score > 1 in both TNBC cells. Further, the P5 predictively targeted the protein kinases that significantly correlated to breast cancer prognosis. The GSEA analysis result shows that receptor tyrosine kinase, Notch3, Notch4, and Ephrin signaling pathways were targeted by P5. The P5 treatment reduced the EGFR expression level and activation in TNBC cells.

## Introduction

Triple-negative breast cancer (TNBC) has the worst prognosis compared to other subtypes of breast cancer. Chemotherapy is the main standard therapy for TNBC patients due to the absence of ER, PR, and HER2 protein expression. Even though TNBC is the most responsive subtype of breast cancer to chemotherapy, chemoresistant is frequently found after TNBC patients are treated with chemotherapy^1^. In the last decade, target therapy research for TNBC cancer patients has been developed rapidly. Atezolizumab was approved by the FDA as the first targeted therapy to treat TNBC patients. This monoclonal antibody targeted the PD–L1 protein in metastatic TNBC patients. Thus inhibiting tumor immune system inactivation leads to immune activation to eliminate the TNBC cells. However, only approximately 40% of TNBC patients are eligible for immunotherapy^2^. Besides immunotherapy, targeting protein kinase provides other potential targeted therapies for TNBC patients. Based on omics study, protein kinase alterations were frequently found in TNBC patients. Previous studies found that protein kinases induce the TNBC cells’ aggressiveness and may provide new potent targeted therapy for TNBC patients^2–4^. Some protein kinase inhibitors have been used in clinical trials to treat TNBC patients such as NBE-002, trastuzumab, ipatasertib, capivasertib, palbociclib, ribociclib, AZD8186, and avastin^5^. Finding a new protein kinase inhibitor to provide a more potent targeted therapy candidate for TNBC patients option is important.

In the previous study, we found a small compound, *N*-phenyl pyrazoline inhibits one of the protein kinases, EGFR leads to cervical cancer aggressiveness attenuation^6^. *N*-phenyl pyrazoline also inhibits the cell viability of human breast cancer cells and colorectal cancer cells^7^. However, the anti-cancer activity and protein kinase inhibition activity of *N*-phenyl pyrazoline in TNBC remain unknown. In this study, we investigated the anti-cancer activity as well as protein kinase inhibition potency of *N*-phenyl pyrazoline in TNBC cells.

## Results

### *N*-phenyl pyrazoline 5 inhibits the TNBC cells’ viability in a dose-dependent manner

**Figure 1:**
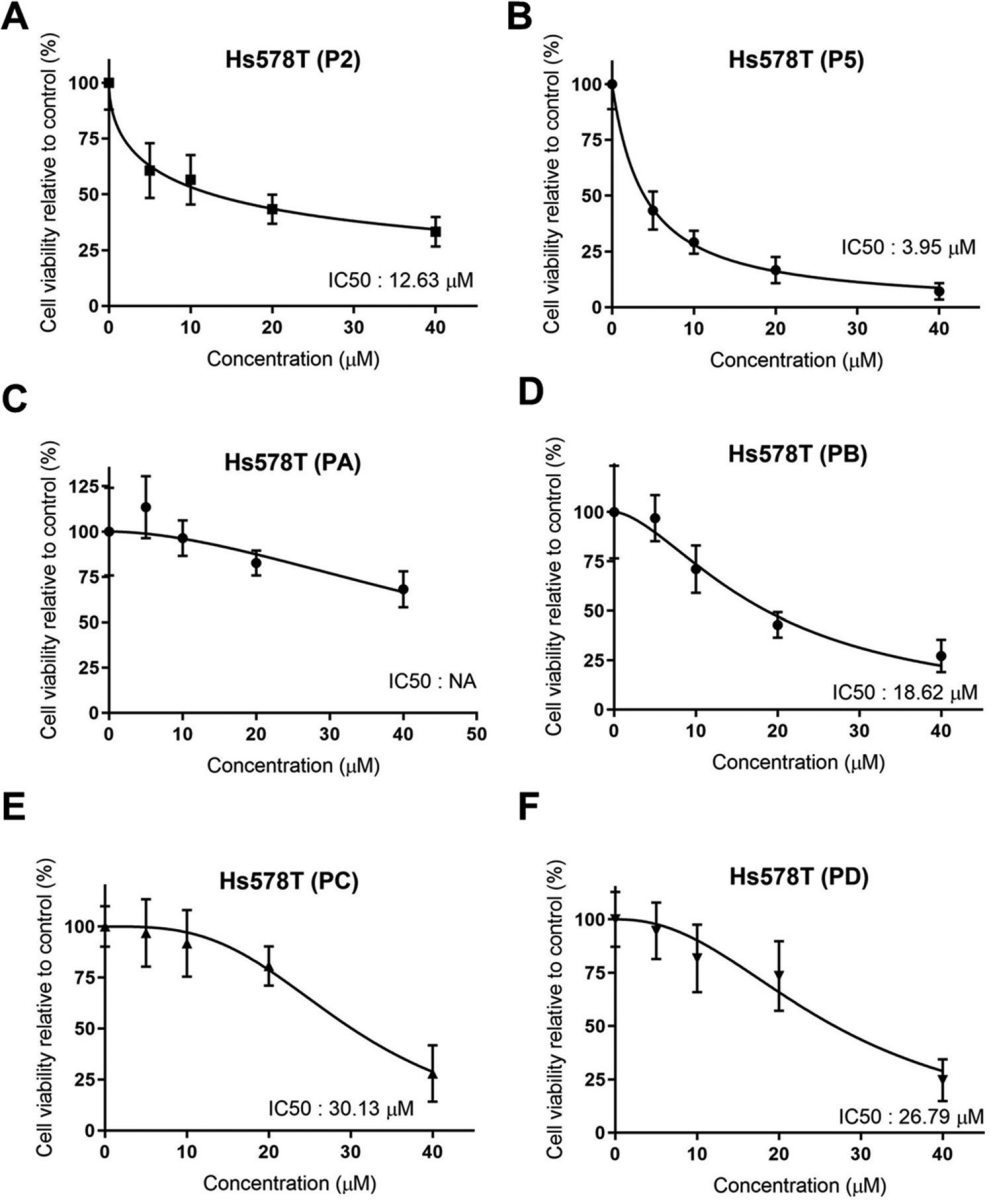
The *N*-phenyl pyrazoline 5 exhibits the most cytotoxic effect against Hs578T cells. Based on MTT assay results, the *N*-phenyl pyrazoline 2, 5, A, B, C, and D inhibits the cell viability of Hs578T cells with IC50 concentrations are 12.63 µM (**A**), 3.95 µM (**B**), NA (**C**), 18.62 µM (**D**), 30.13 µM (**E**), and 26.79 µM (**F**) respectively. NA, not available.

To evaluate the cytotoxicity of *N*-phenyl pyrazoline derivatives, we performed an MTT assay using TNBC cell line, Hs578T cells. We found that P2 and P5 exhibited good activity against Hs578T cells with IC50 12.63 µM, and 3.95 µM respectively (Fig. 1A, B). Meanwhile, cytotoxic effects of the PB, PC, and PD were categorized as moderate activity with IC50 18.62 µM, 30.13 µM, and 26.79 µM respectively (Fig. 1D–F). The IC50 concentration of PA was not available due to the highest concentration used in this study could not suppress the 50% of Hs578T cells viability (Fig. 1C). Further, only the P5 exhibited the IC50 (21.55 µM) against the MDA MB 231 cells (Supplementary Fig. 1 A–F). Meanwhile, the P2, PA, PB, PC, and PD could not suppress the 50% cell viability of MDA MB 231 even after being treated with the highest concentration (40 µM). Later, all *N*-phenyl pyrazoline derivatives inhibit the Hs578T and MDA MB 231 cells’ viability in dose-dependent manners. The P5 is the most potent *N*-phenyl pyrazoline derivative with the lowest IC50 concentration against both TNBC cells. Later, the P5 was used for further study.

### *N*-phenyl pyrazoline 5 attenuates TNBC cell proliferation and migration ability in a dose-dependent manner

**Figure 2:**
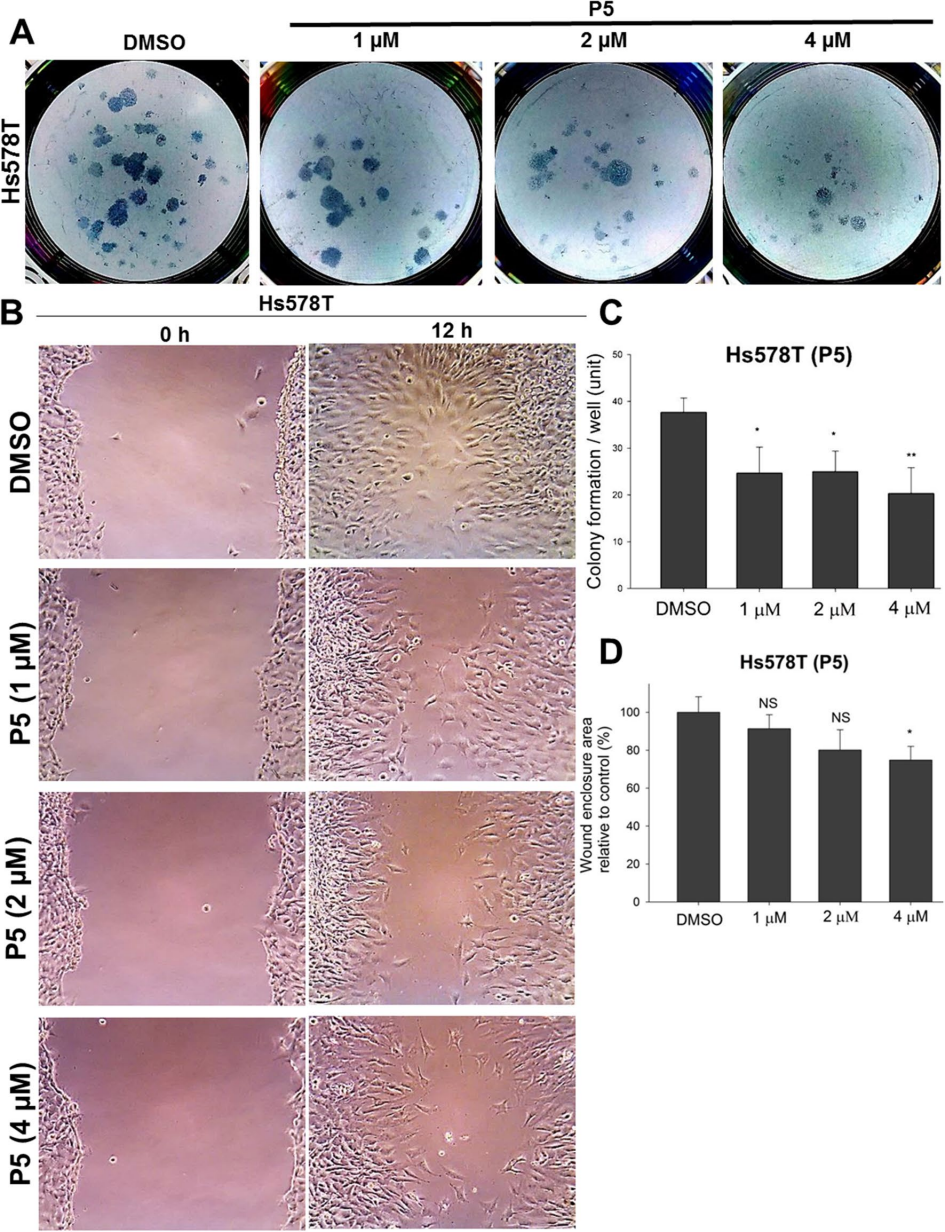
The *N*-phenyl pyrazoline 5 attenuates the cell proliferation and cell migration of Hs578T cells in a dose-dependent manner. (**A**) The colony formation of Hs578T cells is inhibited by *N*-phenyl pyrazoline 5 treatment (1 µM, 2 µM, and 4 µM) after 14 days of incubation. (**B**) The migration ability of Hs578T cells are reduced by *N*-phenyl pyrazoline 5 treatment after 12 h incubation. (**C**) The quantification graphs of colony formation assay results show that P5 treatment significantly reduces the colony number of Hs578T cells. Meanwhile, (**D**) the P5 treatment significantly inhibits the wound enclosure area in the highest concentration group (4 µM). Results expressed as mean ± SD of assays performed at least in triplicate. *p < 0.05, **p < 0.01, ***p < 0.001. Abbreviation: NS, not significant.

To investigate whether the P5 compound inhibits the Hs578T and MDA MB 231 cell proliferation, we performed colony formation assay. The P5 treatment with 1 µM, 2 µM, and 4 µM concentrations markedly inhibited the cell colony numbers of Hs578T cells (Fig. 2A). Statistical calculation showed that P5 treatment with 1 µM, 2 µM, and 4 µM concentrations significantly reduced the cell colonies of Hs578T cells (Fig. 2C). The P5 also inhibited the cell colony formation of MDA MB 231 cells (Supplementary Fig. 2B). The 5 µM, 10 µM, and 20 µM of P5 treatment significantly reduced the cell colony numbers of MDA MB 231 cells (Supplementary Fig. 2D). In the paclitaxel and P5-paclitaxel combination groups, the Hs578T cells had no cell colony observed (Supplementary Fig. 2A and 2D). Meanwhile, in MDA MB 231 cells, the combination of P5 and paclitaxel exhibited lower cell colony numbers than P5 alone or paclitaxel alone (Supplementary Fig. 2B and 2D). These suggest the P5 and paclitaxel may have synergistic effects against TNBC cells. Further, the P5 treatment attenuated the cell migration ability of Hs578T cells (Fig. 2B). Even though the migrated areas (wound enclosure areas) decreased after Hs578T cells were treated with 1 µM, 2 µM, and 4 µM concentrations of P5, only 4 µM concentrations of P5 reduced the migrated area significantly (Fig. 2D). These findings suggest the P5 has an anti-proliferative and anti-migratory effect against Hs578T and MDA MB 231 cells in dose-dependent manners.

### *N*-phenyl pyrazoline 5 suppresses cancer stemness and enhances paclitaxel sensitivity of TNBC cells

**Figure 3:**
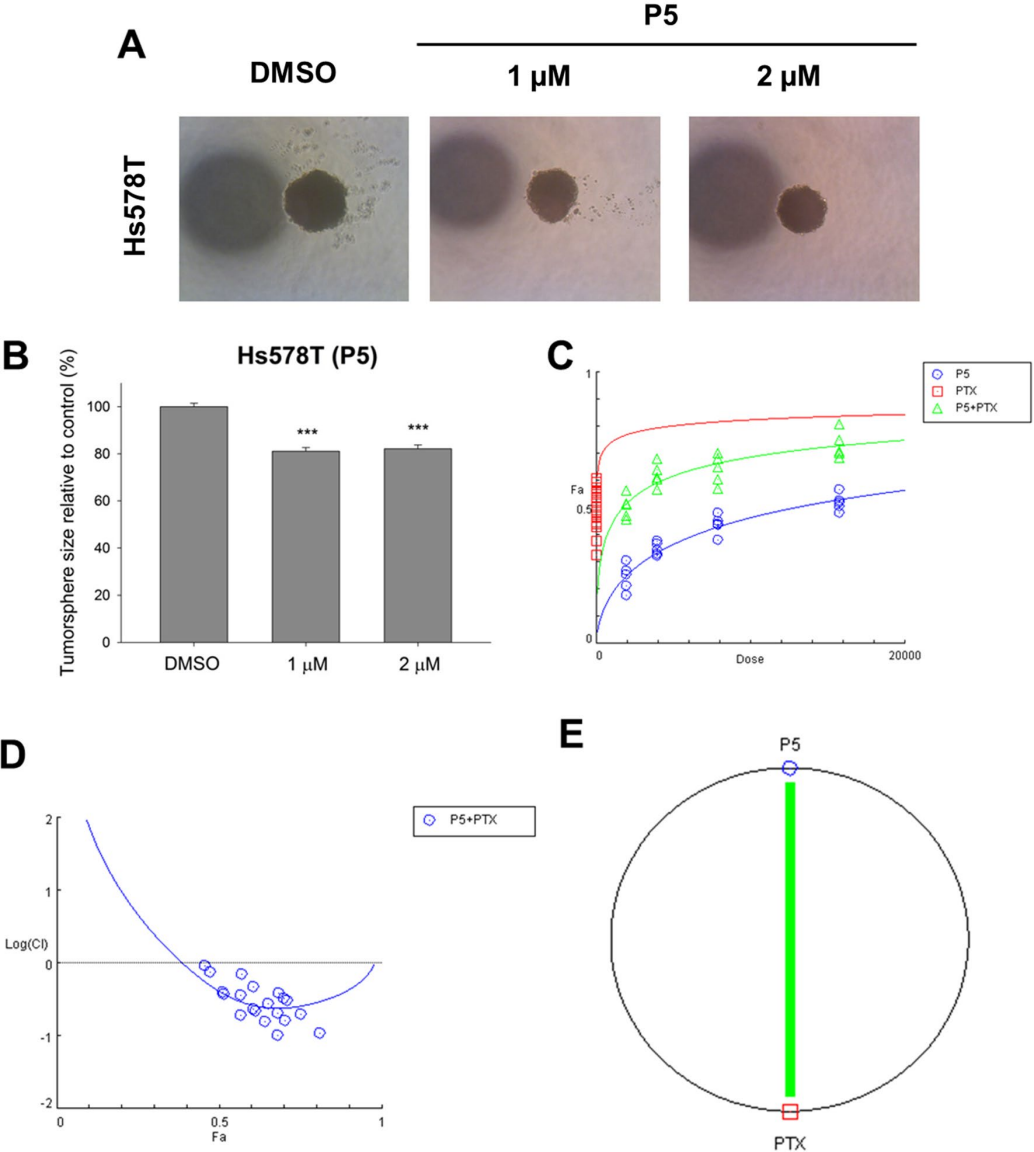
The *N*-phenyl pyrazoline 5 treatment attenuates the cancer stemness of Hs578T cells. (**A**) The P5 reduces the tumorsphere size of Hs578T cells in dose dose-dependent manner. (**B**) Statistical calculation shows that P5 significantly reduces the tumorphere size. (**C**) The combination treatment between P5 and paclitaxel exhibits a synergistic effect. (**D**) The combination index scores are less than 1 for P5 and paclitaxel combination treatments against Hs578T cells. The polygonogram shows a solid green line indicating the synergistic effect. Results expressed as mean ± SD of assays performed at least in triplicate. *p < 0.05, **p < 0.01, ***p < 0.001. Abbreviation: NS, not significant.

To investigate the effect of P5 treatment on the cancer stemness of Hs578T cells, we performed hanging drop tumorsphere assay. We found that treatment of P5 (1 µM, and 2 µM) significantly reduced tumorsphere size of Hs578T cells (Fig. 3A, B). Further, the combination treatment between P5 and paclitaxel exhibited a synergistic effect in Hs578T and MDA MB 231 cells. All combination scores were lower than 1 in the Hs578T cells (Fig. 3C, D). Meanwhile, the combination scores between P5 and paclitaxel in all concentration combinations against MDA MB 231 were lower than 1 except for the lowest concentration (Supplementary Fig. 3A and 3B). The polygonogram also showed the green solid line indicating that both compounds have a synergistic effect (Fig. 3E and Supplementary Fig. 3C). These findings suggest the P5 treatment could both inhibit the cancer stemness and enhance the paclitaxel sensitivity of TNBC cells.

### *N*-phenyl pyrazoline 5 possibly inhibits the TNBC cells by targeting protein kinase

**Figure 4:**
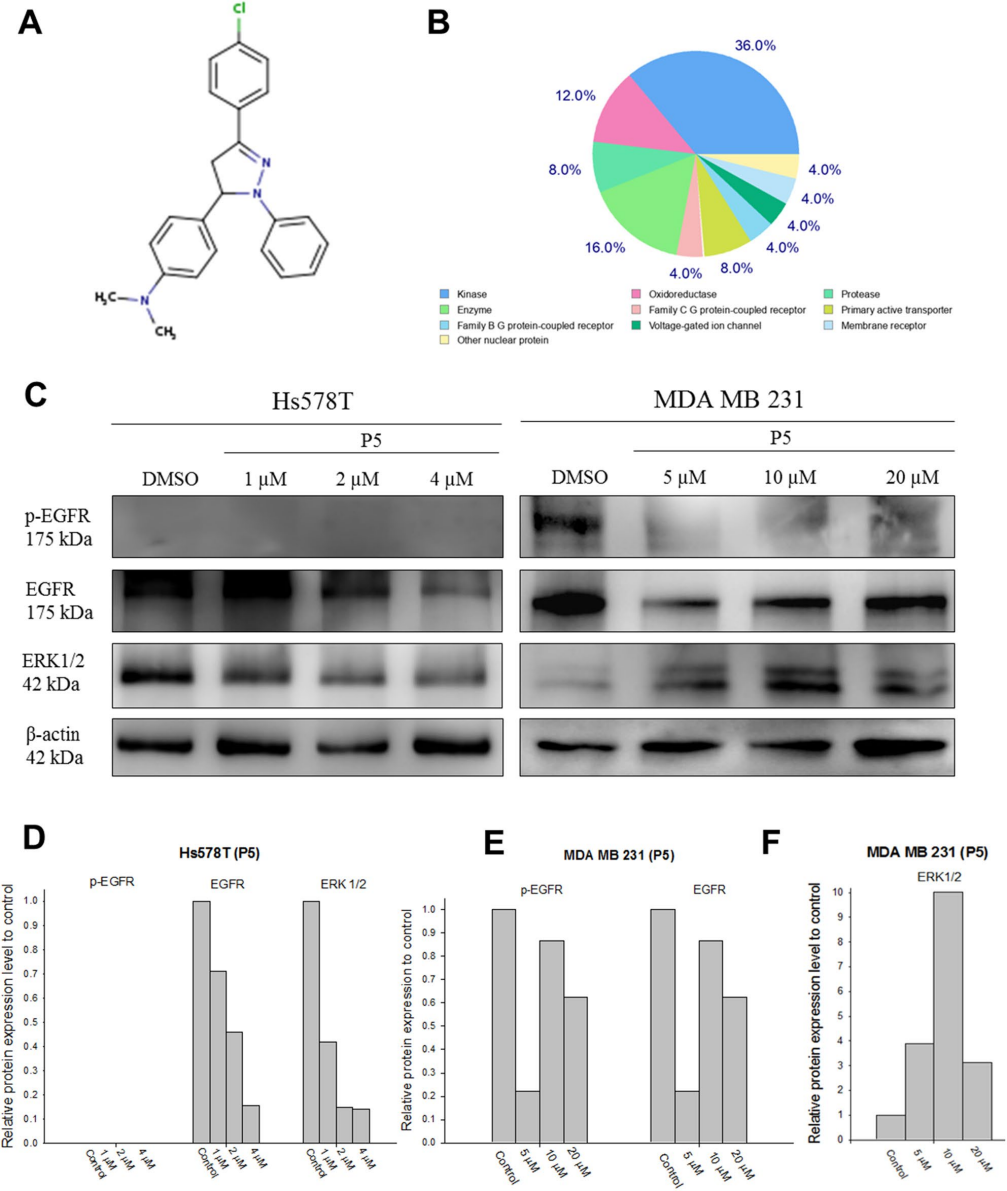
The P5 is predictively bound to the tyrosine kinase proteins. (**A**) Chemical structure of P5. (**B**) The SwissTargetPrediction analysis result shows the protein kinase as the most protein targeted by P5 (36%). (**C**) Western blotting results show the P5 treatment reduced the EGFR expression level in Hs578T and MDA MB 231 cells. The P5 deactivates the EGFR in MDA MB 231 cells. The ERK 1/2 expression levels are affected by the P5 treatment in both TNBC cells. (**D**, **E**, **F**) The quantification graph of p-EGFR, EGFR, ERK 1/2, and β-actin protein level form Hs578T and MDA MB 231 cells.

**Figure 5:**
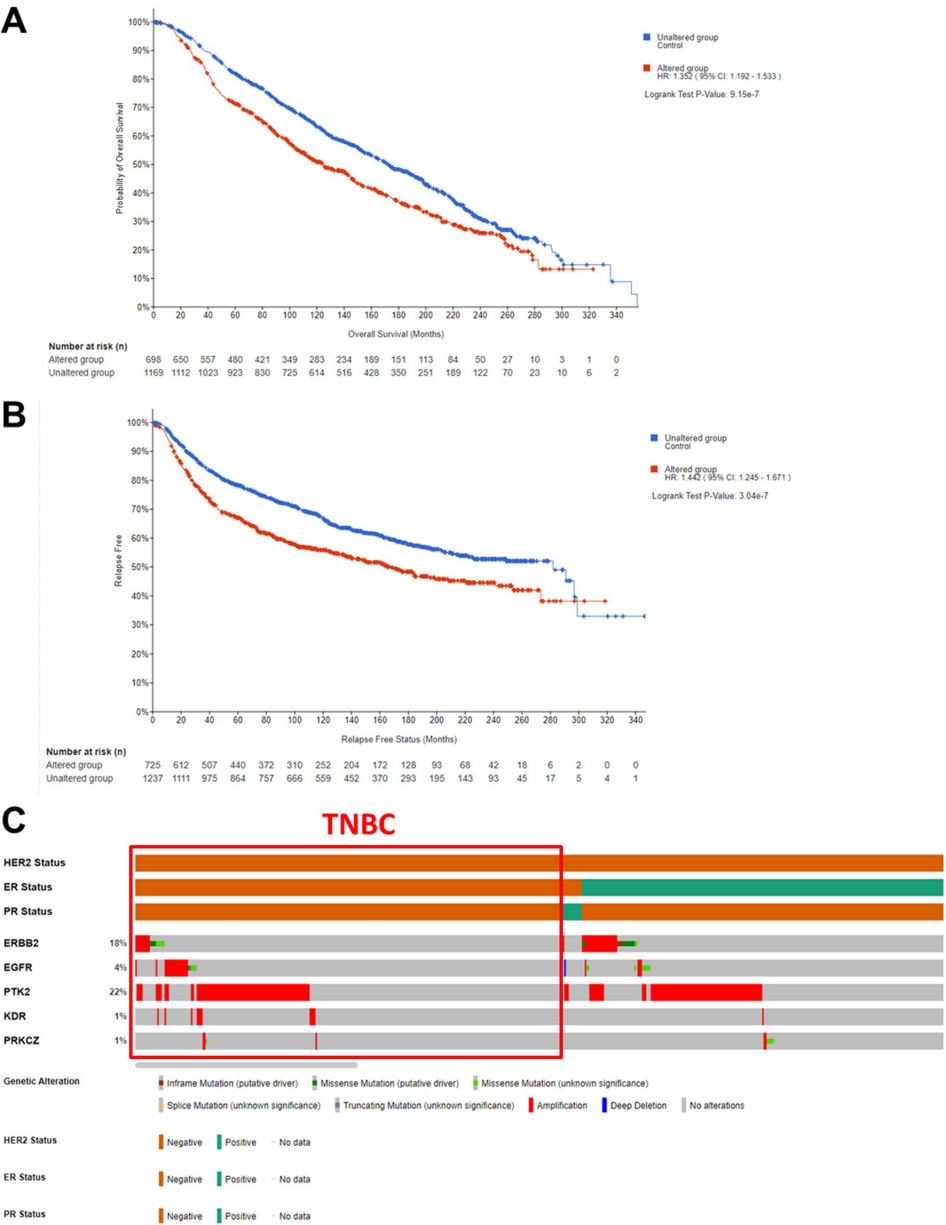
Kaplan–Meier analysis reveals the significant correlations between ERBB2, EGFR, PTK2, KDR, and PRKCZ alterations with (**A**) shorter survival time and (**B**) a high rate of disease relapse in breast cancer patients. (**C**) Alteration profile of TNBC patients for ERBB2, EGFR, PTK2, KDR, and PRKCZ. The PTK2 has the most frequent alteration in TNBC patients.

To investigate the potent protein target of the P5 compound, we used SwissTargetPrediction website platform. Using P5 SMILES data, we found that most (36%) of the predictive target protein of P5 was protein kinase (Fig. 4A, B). The list of all target proteins is provided in Supplementary Table 1. Further, the GSEA analysis results showed the receptor tyrosine kinase signaling pathway is the most significant predictive signaling pathway targeted by P5 (Table 1). Later, to investigate the correlation between target protein expression level and breast cancer patients’ prognosis we used the cBioprtal website platform based on the top 5 target proteins with the highest probability. The list of 5 target proteins is provided in Table 2. The breast cancer patients with alteration of target proteins significantly exhibited a shorter survival time and higher incidence of disease relapse (Fig. 5A, B). These target protein alterations are also frequently found in TNBC patients (Fig. 5C). Our findings suggest that the P5 may inhibit the Hs578T and MDA MB 231 cells by targeting the tyrosine kinase pathway. The ERBB2, EGFR, PTK2, KDR, and PRKCZ may serve as P5 potent target proteins in Hs578T and MDA MB 231 cells. Since the EGFR had the highest probability score as the target of P5 and was predominantly found in TNBC patients^8^, we investigated the P5 effect on EGFR level expression and activation using western blotting assay. We found that the P5 treatment reduced the EGFR expression level in Hs578T and MDA MB 231 cells (Fig. 4C–E). Consistently, the activated form of EGFR (phosphorylated EGFR) was reduced by P5 treatment in MDA MB 231 cells. However, the phosphorylated EGFR could not observed in all groups of Hs578T cells. Further, the ERK 1/2 expression level was decreased by the P5 treatment in dose dose-dependent manner (Fig. 4C, D). Meanwhile, the ERK ½ expression level was increased after P5 treatment in MDA MB 231 cells (Fig. 4C, F). Our findings suggest that EGFR-ERK 1/2 axis was the protein target of P5 in TNBC cells. This axis provided the mechanism behind the P5 anti-cancer activity against the TNBC cells. However, replications of the western blotting assay to evaluate the EGFR-ERK 1/2 is required to validate this mechanism.

**Table 1:** Predicted pathway targeted by P5 based on GSEA analysis.

No	Gene Set Name	Description	Genes in Overlap (k)	p-value	FDR q-value
1	REACTOME_SIGNALING_BY_RECEPTOR_TYROSINE_KINASES	Signaling by receptor tyrosine kinases	16	5.30E−23	8.97E−20
2	REACTOME_NONCANONICAL_ACTIVATION_OF_NOTCH3	Noncanonical activation of NOTCH3	6	2.44E−18	2.06E−15
3	REACTOME_EPH_EPHRIN_SIGNALING	EPH-Ephrin signaling	9	1.29E−17	7.28E−15
4	REACTOME_EPH_EPHRIN_MEDIATED_REPULSION_OF_CELLS	EPH-ephrin mediated repulsion of cells	8	1.79E−17	7.57E−15
5	REACTOME_NOTCH3_ACTIVATION_AND_TRANSMISSION_OF_SIGNAL_TO_THE_NUCLEUS	NOTCH3 activation and transmission of signal to the nucleus	7	2.43E−17	8.23E−15
6	REACTOME_NOTCH4_ACTIVATION_AND_TRANSMISSION_OF_SIGNAL_TO_THE_NUCLEUS	NOTCH4 activation and transmission of signal to the nucleus	6	4.02E−17	9.72E−15
7	REACTOME_REGULATED_PROTEOLYSIS_OF_P75NTR	Regulated proteolysis of p75NTR	6	4.02E−17	9.72E−15
8	REACTOME_DISEASES_OF_SIGNAL_TRANSDUCTION_BY_GROWTH_FACTOR_RECEPTORS_AND_SECOND_MESSENGERS	Diseases of signal transduction by growth factor receptors and second messengers	12	1.16E−16	2.46E−14
9	REACTOME_NRIF_SIGNALS_CELL_DEATH_FROM_THE_NUCLEUS	NRIF signals cell death from the nucleus	6	6.95E−16	1.31E−13
10	REACTOME_NERVOUS_SYSTEM_DEVELOPMENT	Nervous system development	12	3.91E−15	6.61E−13

**Table 2:** Top 5 predictive proteins targeted by P5 based on SwissTargetPrediction analysis.

No.	Target	Uniprot ID	Target class	Probability
1	ERBB2	P04626	Kinase	0.233302811
2	EGFR	P00533	Kinase	0.233302811
3	PTK2	Q05397	Kinase	0.137288883
4	KDR	P35968	Kinase	0.137288883
5	PRKCZ	Q05513	Kinase	0.12928422

## Discussion

Triple-negative breast cancer is classified as a breast cancer subtype with the absence of estrogen receptor (ER), progesterone receptor (PR), and human epidermal growth factor receptor 2 (HER2). It has the worst clinical prognosis due to aggressive phenotype and rapid disease progression. Targeting therapy studies in TNBC patients have been moved rapidly. After FDA approval of immune checkpoint inhibitor therapy for TNBC patients, recently targeting tyrosine kinase as another optional therapy has been moved from a preclinical study to a clinical trial study to treat TNBC patients. A phase Ib clinical study showed that a combination of a tyrosine kinase inhibitor (anlotinib) and an immune checkpoint inhibitor (anti-PD-L1 antibody) showed promising efficacy with a good safety profile provides a chemotherapy-free treatment for TNBC patients^9^. In another phase II clinical trial, the combination of this tyrosine kinase inhibitor and chemotherapy also showed a certain efficacy and acceptable safety profile to treat metastatic triple-negative breast cancer patients^10^. A tyrosine kinase, SRC overexpression is frequently found in TNBC patients and associated with tumor malignancy. In vitro and in vivo studies showed that tyrosine kinase induces aggressiveness of TNBC cells including cancer stemness, tumor growth, and metastasis^11^. Exploring new tyrosine kinase inhibitors are important to treat TNBC patients. Our *N*-phenyl pyrazoline derivative attenuates the cell viability, cell migration, and cancer stemness of cervical cancer cells via EGFR inhibition^6^. In the present study, to the best of our knowledge, we provide the first evidence of the anti-cancer activity of *N*-phenyl pyrazoline 5 in TNBC cells by inhibiting cell viability, cell proliferation, cell migration, and cancer stemness. Many pyrazoline derivatives showed tyrosine kinase inhibition activity in solid tumors. Thus compounds inhibit the EGFR and VEGFR activity^12,13^. Thus consistent with our present study we found protein kinase/tyrosine kinase was the most predicted target of P5 using in silico study. The ERBB2, EGFR, PTK2, KDR, and PRKCZ were the predicted targets of P5 with the highest probability relative to other targets. The EGFR expression positively correlates with a more aggressive characteristic in TNBC patients^8^. The PTK2 is highly expressed in TNBC patients compared to non-TNBC patients and normal breasts^14^. Consistent with our results, alteration of ERBB2, EGFR, PTK2, KDR, and PRKCZ significantly correlated with shorter survival time and high incidence of disease relapse in breast cancer patients. Further, we confirmed the EGFR expression level and activation were inhibited by P5 treatment in TNBC cells. KRAS mutation and ERK activation were found as the mechanism behind the EGFR inhibitor resistance in cancer cells including in breast cancer. Further, the KRAS mutation was found in MDA MB 231 cells, but not the Hs578T cells^15–17^. Consistent with our finding that MDA MB 231 TNBC cells are less sensitive to P5 treatment targeting EGFR. Further, the ERK 1/2 protein level also increased by EGFR inhibition by P5 treatment.

Based on our findings, *N*-phenyl pyrazoline 5 (P5) is the most potent compound against TNBC cells. The P5 inhibits the cell viability, cell proliferation, cell migration, and cancer stemness of TNBC cells possibly via targeting EGFR-ERK 1/2 signaling pathways (Supplementary Fig. 4). However, replications study is required to validate this mechanism.

## Materials and methods

### Cell culture and materials

In this study, we purchased a human TNBC cell line, the Hs578T cell line from Elabscience Biotechnology Inc. The MDA MB 231 cell was obtained from Professor Sofia Mubarika Haryana (Departement of Histology and Cell Biology, Faculty of Medicine Public Health and Nursing, Universitas Gadjah Mada). The Hs578T cell line was maintained in DMEM medium (#12100046, Thermo Fisher Scientific. Inc) supplemented with 1% Penicillin–Streptomycin, 10% FBS (#Gibco™ 10270106, Thermo Fisher Scientific. Inc), and 0.5% of amphotericin B. The MDA MB 231 cell line was cultured with DMEM medium (#12100046, Thermo Fisher Scientific. Inc) supplemented with 1% Penicillin -Streptomycin, and 10% FBS (#Gibco™ 10270106, Thermo Fisher Scientific. Inc). The culture conditions for both cell lines were carried out in an incubator at 37 °C with 5% CO^2^. The cells were subcultured every 48–72 h for maintenance. The *N*-phenyl pyrazoline derivative compounds were obtained from Professor Tutik Dwi Wahyuningsih’s laboratory. These *N*-phenyl pyrazoline derivative compounds were synthesized as previously described^6^.

### MTT assay

The 5 × 10^3^ Hs578T cells and 1 × 10^4^ MDA MB 231 cells were seeded in each well of 96-well plates. After incubated with basal DMEM medium (#12100046, Thermo Fisher Scientific. Inc), the Hs578T cells were treated with *N*-phenyl pyrazoline 2 (P2), *N*-phenyl pyrazoline 5 (P5), *N*-phenyl pyrazoline A (PA), *N*-phenyl pyrazoline B (PB), *N*-phenyl pyrazoline C (PC), and *N*-phenyl pyrazoline D (PD) compounds. Meanwhile, after incubated with DMEM medium supplemented with 3% of FBS, the MDA MB 231 cells were treated with P2, P5, PA, PB, PC, and PD. The control groups for both cells were treated with DMSO (Merck) with a concentration equal to the highest amount of DMSO (0.04%) used in the treatment groups. After incubation for 48 h, the medium was discharged from each well. The cells were added with 100 µL of 10% 3-(4,5-dimethylthiazol-2yl)-2,5-diphenyltetrazolium bromide (2138419, Invitrogen) and incubated for 4 h in a 5% CO^2^ incubator, at 37 °C. Then 100 µL of 10% SDS (sodium dodecyl sulfate, Merck) was added to the cells in each well. Incubation for overnight was performed in a dark room at room temperature. The absorbance values were quantified using a spectrophotometry at 565 nm wavelength. IC50 analyses were performed using GraphPad Prism software.

### Colony formation assay

The 1 × 10^2^ Hs578T cells and 4 × 10^2^ MDA MB 231 cells were seeded onto 6-well plates. 2 mL of DMEM (#12100046, Thermo Fisher Scientific. Inc) supplemented with 3% FBS (#Gibco™ 10270106, Thermo Fisher Scientific. Inc) was used as a medium for each well of Hs578T cells. While MDA MB 231 used 2 mL of DMEM supplemented with 10% FBS for each well. After being incubated overnight, the cells in control groups were treated with 0.02% DMSO (Merck). The treatment groups were treated with 1 µM, 2 µM, and 4 µM of P5 compound for Hs578T. The MDA MB 231 cells were treated with 5 µM, 10 µM, and 20 µM of P5 compound. To investigate the combination effect of P5 and paclitaxel (Sindaxel, Actavis) on cell proliferation, the Hs578T and MDA MB 231 cells were also treated with P5 alone, paclitaxel alone, and P5-paclitaxel combination. Incubation was carried out for 14 days (Hs578T) and 8 days (MDA MB 231). After incubation, the mediums were discharged. The cells were fixed by adding 2 mL of ice-cold methanol absolute. After 15 min of methanol incubation at − 20 °C, the ethanol was discharged and the cells were washed with PBS (2219206, Gibco). For cell staining, 1% of methylene blue diluted in methanol was added to each well and incubation was carried out for 4 h at room temperature. After 1% methanol was discharged, the cells were washed with PBS. The cells were dried by putting the plate at room temperature. The colonies were observed under a microscope.

### Migration assay

The 1.25 × 10^4^ Hs578T cells were seeded onto 24-well plates for each well using a basal DMEM medium (#12100046, Thermo Fisher Scientific. Inc) without any supplementation overnight. The approximately 100% monolayer confluent cells in the well were used for migration assay. The scratch was made using a sterile yellow pipette tip. After the medium was discharged, the cells were washed with PBS (2219206, Gibco) to remove unattached cells. The 0.004% DMSO (Merck) was used to treat the control group. Treatment groups were treated with P5 compound (1 µM, 2 µM, and 4 µM). To evaluate the anti-migrative effect of P in Hs578T cells, images of each well from all groups were captured at 0 and 12 h after the treatment initiation. The migration cell areas were quantified using ImageJ version 1.518j software (Wayne Rasband NIH, Bethesda, MD, USA). The migrated area quantification was performed by measuring the 0 h gap area and 12 h gap area for each well. The migrated area was obtained by subtraction (migrated area (unit) = 0 h gap area—12 h gap area).

### Hanging-drop tumorsphere assay

The confluence Hs578T cells were detached from the culture dish using trypsin and were collected. Later, the cells were resuspended in a complete growth medium (2.5 cells × 10^3^ cells/20 µL). The cells were divided into control and treatment groups. The 0.004% DMSO (Merck) was used to treat the control group cells, while the treatment group cells were treated with different concentrations of *N*-phenyl pyrazoline (1 µM, 2 µM, and 4 µM). Five ml PBS (2,219,206, Gibco) was added to a 100 mm culture dish. Later, the 20 µL of cell suspension from each group was seeded in the inner side of the 100 mm culture dish inner side. The lid was flipped gently and put back into the bottom part of the dish culture that had already been added with PBS. After 7 days of incubation in a 5% CO^2^ incubator at 37 °C, the tumorspheres were observed and images were taken under the microscope. Tumorsphere size was quantified using ImageJ version 1.51j8 (Wayne Rasband NIH, Bethesda, MD, USA).

### Drug combination index

To evaluate whether the P5 treatment enhanced the Hs578T and MDA MB 231 cells’ sensitivity to paclitaxel, we performed a drug combination assay. The constant drug ratio combination 1:1000 for paclitaxel (Sindaxel, Actavis) and P5 combination treatment against Hs578T cells. The constant drug ratio combination is 1:250 for paclitaxel and P5 combination treatment against MDA MB 231 cells. Drug combination index scores were analyzed using CompuSyn software. The synergistic effect was determined by CI score > 1, while the antagonist effect was determined by CI score > 1. The CI score equal to 1 was determined for the addictive effect as described previously^18^.

### Protein and signaling pathway target prediction analysis

The SMILES data of *N*-phenyl pyrazoline 5 was prepared and was used to predict its protein target using the SwissTargetPrediction platform (http://www.swisstargetprediction.ch/). Based on predicted protein targets of P5, the signaling pathway prediction analysis was carried out using the GSEA platform (https://www.gsea-msigdb.org/gsea/index.jsp0). To evaluate the correlation between predicted target protein and breast cancer patient prognosis, we performed a Kaplan–Meier survival analysis using cBioportal website platform.

### Western blotting assay

The control and treated groups of Hs578T and MDA MB 231 cells were harvested using the cell scraper. The 1X RIPA lysis buffer (ab156034, Abcam) was added to harvested cells and sonication was carried out to break the cells. Later, the cell mixtures were incubated for 30 min on ice. The 12.000 rpm centrifugation at 4 °C was performed and supernatants were carefully collected as total protein samples for each group. Protein quantification was performed using Pierce ™ BCA Protein Assay Kit (23227, Thermo Fisher Scientific). After being added with sample buffer, each sample was heated at 90 °C for 10 min. The 12.000 rpm centrifugation was performed and the samples were loaded onto each SDS-PAGE gel well. The SDS-PAGEs were run and the blots were transferred onto nitrocellulose membrane. Later, the nitrocellulose membranes were incubated with EveryBlot Blocking Buffer (Bio-Rad) for 1 h at room temperature. After being washed with PBST, the membranes were incubated with primary antibody against pEGFR (AP0994, Abclonal), EGFR (A11352, Abclonal), ERK1/2 (A16686, Abclonal), and β-actin (AC026, Abclonal) at 4 °C for overnight. After overnight incubation, the membranes were washed with PBST and incubated with the secondary antibody (AS014, Abclonal) at room temperature for 1 h. The protein visualization was carried out using the UVP Biospectrume Imaging System with SuperKinase TM West Femto Maximum—ECL reagent (BUM102-EN, Abbkine)^6^.

### Statistical analysis

The in vitro assays were carried out at least three times, and results were shown as mean ± SD. Data quantification of migration assay, colony formation assays, hanging-drop tumorsphere assay, and western blotting assay were performed using ImageJ version 1.51j8 (Wayne Rasband NIH, Bethesda, MD, USA). The MTT assay data were analyzed using GraphPad Prism Sofware. Statistical p-value calculations were performed and were visualized using SigmaPlot software.

### Supplementary Information

Supplementary Figure 1.

Supplementary Figure 2.

Supplementary Figure 3.

Supplementary Figure 4.

Supplementary Table 1.

## Data Availability

Data will be made available on request by contacting the corresponding author.
