# Supplementary figures and images for: N-phenyl pyrazoline derivative inhibits cell aggressiveness and enhances paclitaxel sensitivity of triple negative breast cancer cells
Source: Sci Rep. 2024 Jun 8;14:13200. doi: 10.1038/s41598-024-63778-2 (PMC11162478; doi:10.1038/s41598-024-63778-2)

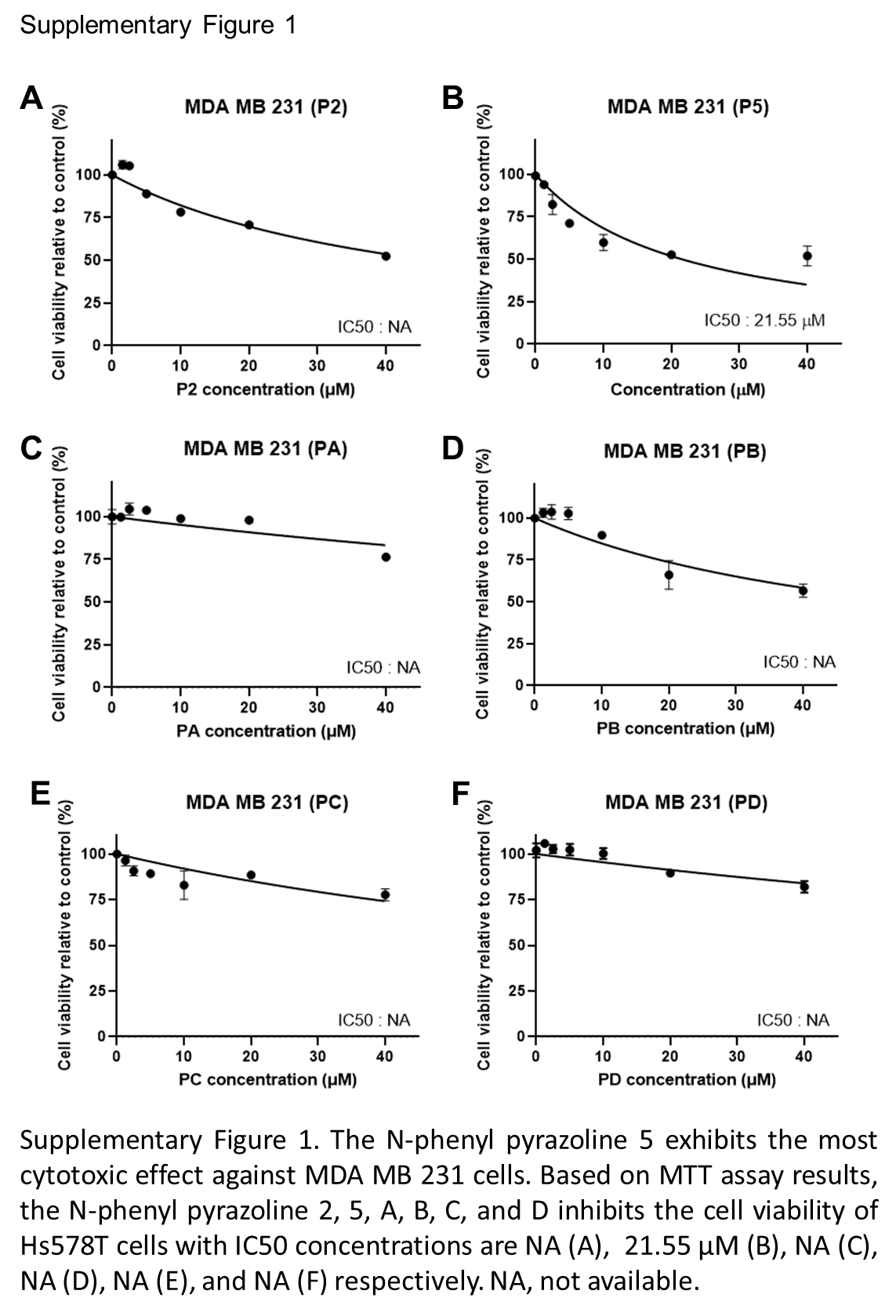

Supplement: Supplementary file 1 — Supplementary Figure 1. [file 41598_2024_63778_MOESM1_ESM.tif]

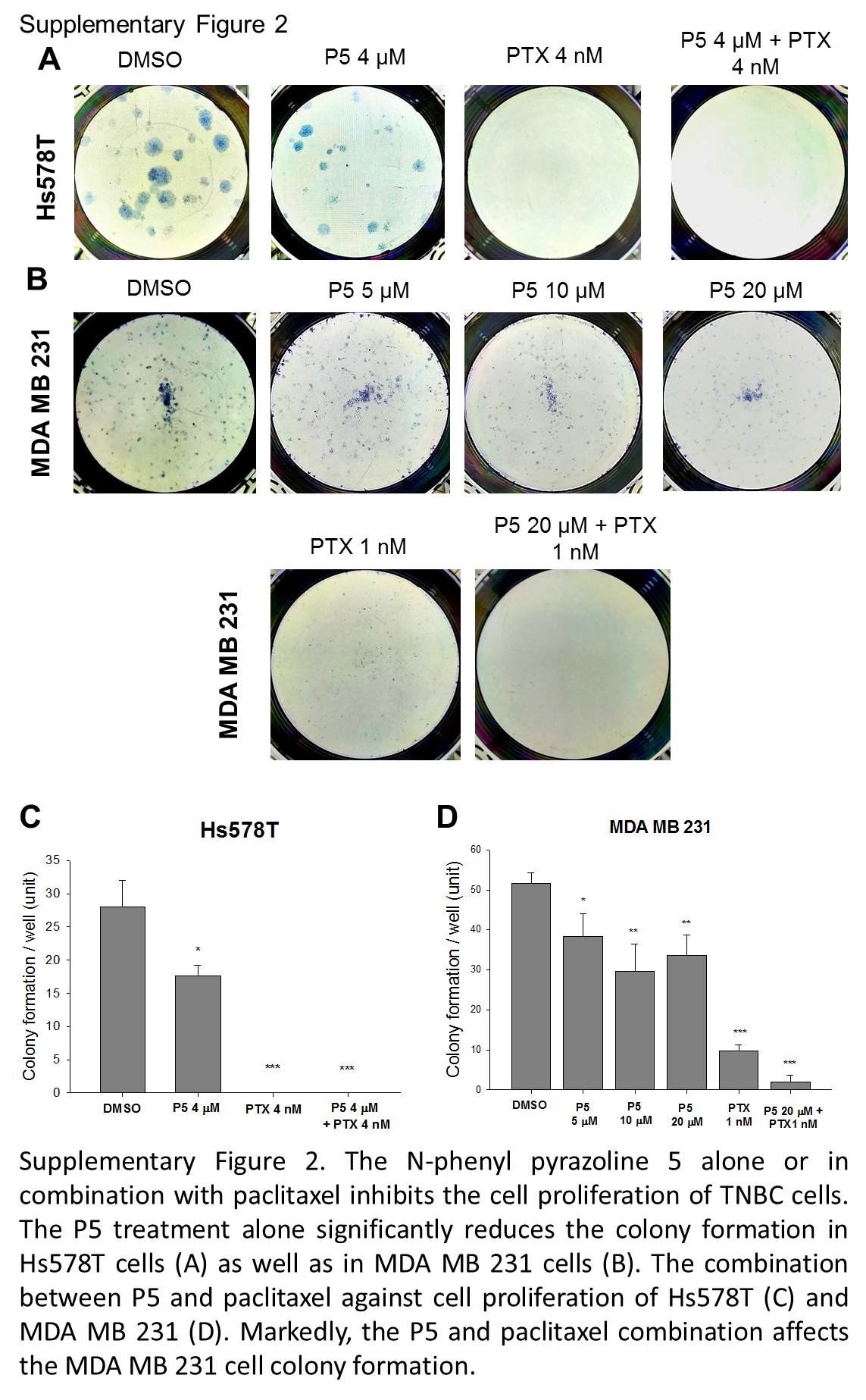

Supplement: Supplementary file 2 — Supplementary Figure 2. [file 41598_2024_63778_MOESM2_ESM.tif]

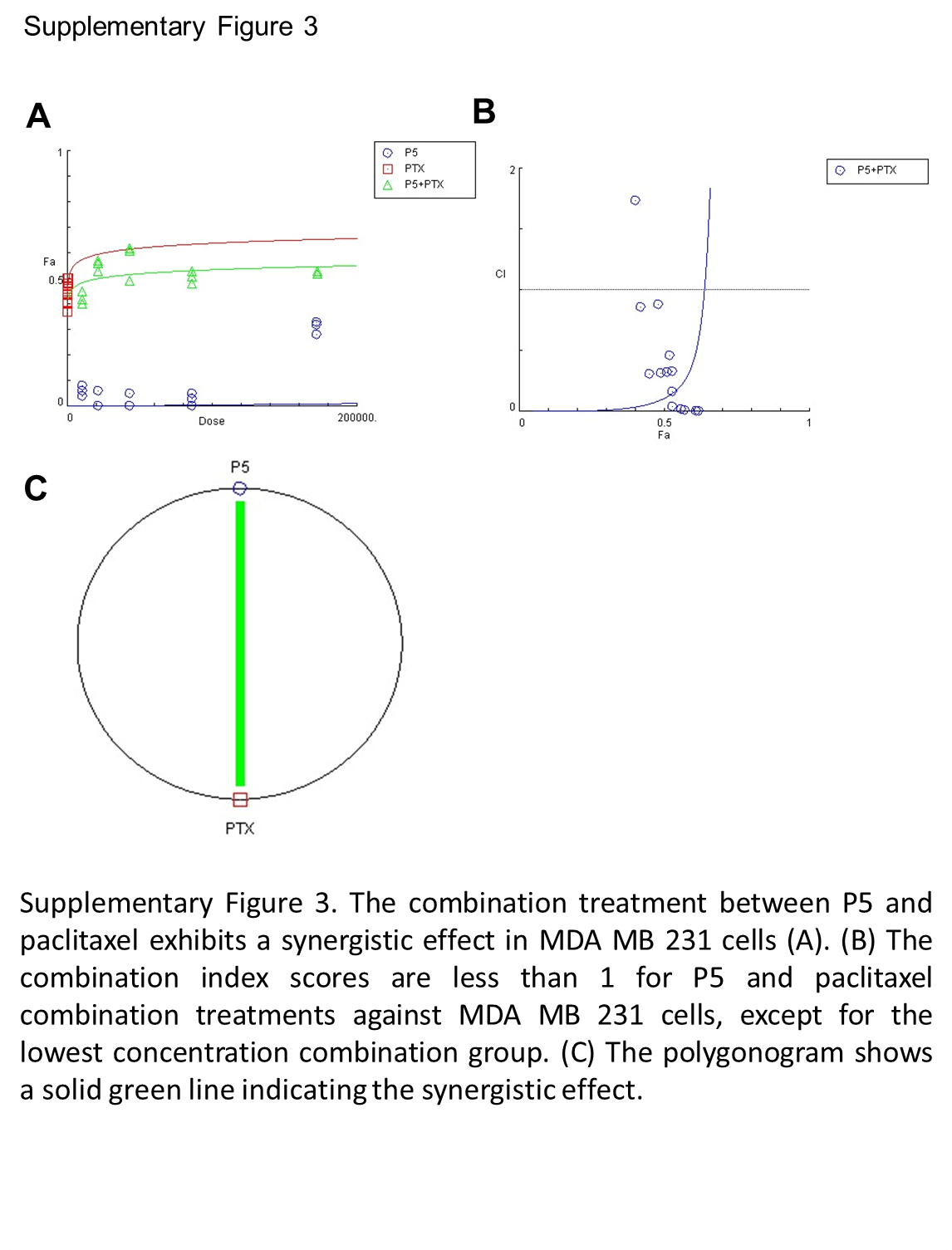

Supplement: Supplementary file 3 — Supplementary Figure 3. [file 41598_2024_63778_MOESM3_ESM.tif]

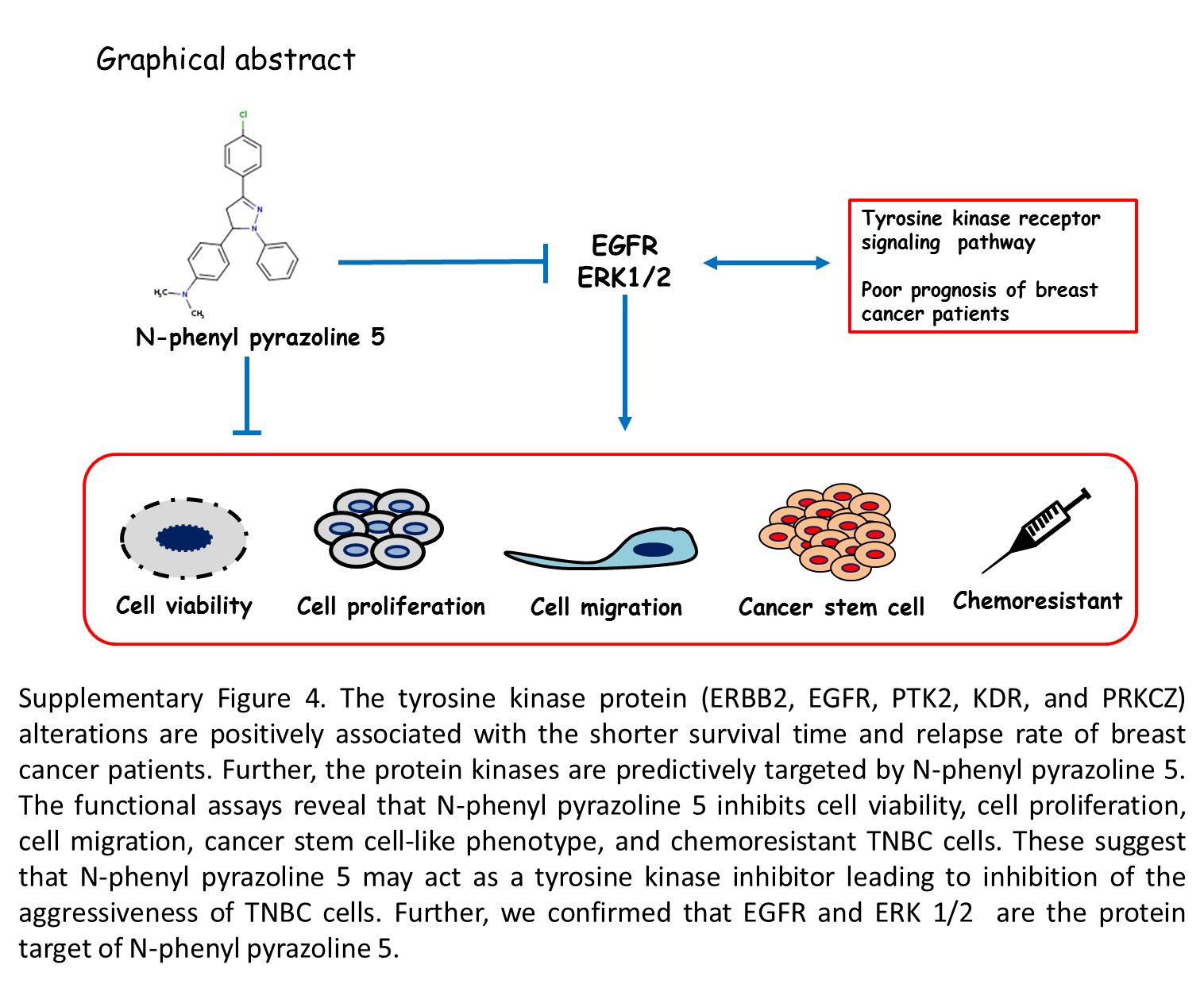

Supplement: Supplementary file 4 — Supplementary Figure 4. [file 41598_2024_63778_MOESM4_ESM.tif]
