# Supplementary material for: N-phenyl pyrazoline derivative inhibits cell aggressiveness and enhances paclitaxel sensitivity of triple negative breast cancer cells
Source: Sci Rep. 2024 Jun 8;14:13200. doi: 10.1038/s41598-024-63778-2 (PMC11162478; doi:10.1038/s41598-024-63778-2)
Supplement: Supplementary file 5 — Supplementary Table 1. [file 41598_2024_63778_MOESM5_ESM.docx]

**Supplementary Table 1.** All predictive proteins targeted by P5

| Target | Common name | Target Class | Probability* |
| --- | --- | --- | --- |
| Receptor protein-tyrosine kinase erbB-2 | ERBB2 | Kinase | 0.233302811 |
| Epidermal growth factor receptor erbB1 | EGFR | Kinase | 0.233302811 |
| Focal adhesion kinase 1 | PTK2 | Kinase | 0.137288883 |
| Vascular endothelial growth factor receptor 2 | KDR | Kinase | 0.137288883 |
| Protein kinase C (PKC) | PRKCZ | Kinase | 0.12928422 |
| Cyclooxygenase-2 | PTGS2 | Oxidoreductase | 0.113285953 |
| Matrix metalloproteinase 9 | MMP9 | Protease | 0.113285953 |
| Matrix metalloproteinase 2 | MMP2 | Protease | 0.113285953 |
| Nitric oxide synthase, inducible (by homology) | NOS2 | Enzyme | 0.113285953 |
| Metabotropic glutamate receptor 2 | GRM2 | Family C G protein-coupled receptor | 0.113285953 |
| Aldose reductase (by homology) | AKR1B1 | Enzyme | 0.113285953 |
| Transitional endoplasmic reticulum ATPase | VCP | Primary active transporter | 0.113285953 |
| Corticotropin releasing factor receptor 1 | CRHR1 | Family B G protein-coupled receptor | 0.113285953 |
| HERG | KCNH2 | Voltage-gated ion channel | 0.113285953 |
| ATP-binding cassette sub-family G member 2 | ABCG2 | Primary active transporter | 0.113285953 |
| Translocator protein (by homology) | TSPO | Membrane receptor | 0.113285953 |
| Monoamine oxidase A | MAOA | Oxidoreductase | 0.113285953 |
| Monoamine oxidase B | MAOB | Oxidoreductase | 0.113285953 |
| p53-binding protein Mdm-2 | MDM2 | Other nuclear protein | 0.113285953 |
| Nerve growth factor receptor Trk-A | NTRK1 | Kinase | 0.113285953 |
| Neurotrophic tyrosine kinase receptor type 2 | NTRK2 | Kinase | 0.113285953 |
| NT-3 growth factor receptor | NTRK3 | Kinase | 0.113285953 |
| Aldo-keto-reductase family 1 member C3 | AKR1C3 | Enzyme | 0.113285953 |
| Serine/threonine-protein kinase B-raf | BRAF | Kinase | 0.113285953 |
| Gamma-secretase | PSEN2 PSENEN NCSTN APH1A PSEN1 APH1B | Protease | 0.113285953 |
| Sodium channel protein type IX alpha subunit | SCN9A | Voltage-gated ion channel | 0.113285953 |
